# Resilience and social support of young adults living with mental illness in the city of Tshwane, Gauteng province, South Africa

**DOI:** 10.4102/curationis.v43i1.2084

**Published:** 2020-12-18

**Authors:** Nok’khanya F. Hadebe, Tendani S. Ramukumba

**Affiliations:** 1 Adelaide Tambo School of Nursing Science, Faculty of Science, Tshwane University of Technology, Pretoria, South Africa

**Keywords:** resilience, social support, young adults, mental illness, family support, supportive relationships

## Abstract

**Background:**

Young adults living with mental illnesses often experience a flood of powerful negative emotions, including anger, anxiety and depression. Some of these young adults remain trapped in their negative emotions long after the stressful events that caused them have passed, whilst resilient young adults without mental illness are able to quickly bounce back to their normal emotional state.

**Objectives:**

The objective of the study was to explore the social supports of young adults living with mental illness in the city of Tshwane.

**Methods:**

This was a qualitative explorative study conducted in the city of Tshwane in 2018 amongst young adults living with mental illness, using a semi-structured interview schedule.

**Results:**

Those young adults living with mental illness who had support from family and friends were able to cope with stressful challenges and had a better outlook for the future, whilst those who perceived their relationship with friends and family as not supportive reported low self-esteem and difficulties dealing with challenging and stressful situations in their lives.

**Conclusion:**

Resilience was seen in those young people living with mental illness with support from family and friends, who had positive future prospects, those with high self-esteem and those who were able to adapt to changing situations beyond their control.

## Introduction and background

Mental illnesses rank third in their contribution to the burden of disease in South Africa, and in 2012, approximately one in six South Africans experienced a common mental illness such as depression, anxiety or substance use illness (Lund et al. 2012:402). Globally, 264 million people suffer from depression, 50 million suffer from dementia, 45 million suffer from bipolar and 20 million suffer from schizophrenia and psychosis (World Health Organization [WHO] 2019). Mental illnesses are characterised by a clinically significant disturbance in cognition, emotion regulation or behaviour that reflects a dysfunction in the psychological, biological or developmental processes underlying mental functioning (Aftab 2016:11). Mental health, on the contrary, is a state of well-being in which every individual realises his or her own potential, can cope with the normal stresses of life, can work productively and fruitfully and is able to contribute to her or his community (WHO 2014).

Mental illnesses are often accompanied by discrimination. Young adults who already experience prejudice and discrimination, in particular, suffer doubly when faced with the burdens of mental illnesses (Collins et al. 2012:2). They face demands, expectations and challenges that carry larger risks than those experienced by their peers some generations ago (Rowling 2006:101). Poor mental health is related to other health and development concerns in young people, such as lower educational achievements, substance abuse, violence, and poor reproductive and sexual health (Sharan & Sagar 2007). Although they are often first detected later in life, several mental illnesses have their onset at ages 14–35 years (WHO 2001). This is the population with the highest prevalence of mental health illnesses, particularly those young adults aged between 18 and 24 years (Rowling 2006:101).

The negative experiences of this group create vulnerabilities and exert certain constraints in access to resources, triggering mental illnesses (Rowling 2006:101), which are usually associated with significant distress in social, occupational or other important activities (Maisel 2013:1). These negative experiences not only affect their mental health but also limit their capacity to acquire the skills to feel confident in interpersonal interactions and resilience in case of life stresses (Rowling 2006:102). Some of the most important determinants of mental illnesses include lack of social support and social protection (WHO 2019).

Resilience is the ability of an individual to function completely in the face of adversity or stress, and it is considered one of the dimensions of positive mental health (Murphey, Barry, & Vaughn 2013:1). Resilience is a combination of personal characteristics and some learned skills (International Council of Nurses 2016:31). This concept generally refers to the capacity of people who are faced with adversity to adapt, cope, rebound, withstand, grow, survive and define a new sense of self through situations of adversity, including mental illness (Deegan 2005:1). Young adults who are resilient are likely to enter adulthood with a good chance of coping well when experiencing difficult circumstances in life (Murphey et al. 2013:1). Young adults with mental illnesses often experience a flood of powerful negative emotions, such as anger, anxiety and depression. Some remain trapped in these negative emotions long after the stressful events that caused them have passed, whilst emotionally resilient young adults have the ability to bounce back to their normal emotional state (Mills & Dombeck 2005).

The demands, expectations, challenges, experiences and circumstances faced by young adults not only affect their mental health but also limit their capacity to acquire the skills to feel confident in interpersonal interactions and resilience in case of challenges (Rowling 2006:102). These experiences continue to put them at risk of social exclusion and discrimination in all facets of life (Murphey et al. 2013:4). Furthermore, the status quo leads to young adults living with mental illnesses being unable to exhibit resilience. Supporting individuals as they develop their own resilience is of significant benefit to individuals, families and organisations (International Council of Nurses 2016:31).

Previous research has indicated associations between social support and resilience in mental health through several psychological and behavioural mechanisms such as the motivation to adopt a healthy lifestyle, high self-esteem and the use of active coping strategies, amongst others (Southwick et al. 2016:78). Therefore, this article reports on the social support of young adults living with mental illnesses.

## Objective of the study

The objective of the study was to explore the social support of young adults living with mental illnesses in the city of Tshwane, Gauteng Province, South Africa.

## Research methods and design

### Research design

A qualitative exploratory research approach was followed in this study. The purpose of qualitative research is to explore a phenomenon from the participant’s point of view (De Vos et al. 2012:64), whilst exploratory studies are conducted to gain insight into a situation, phenomenon, community or individual (De Vos et al. 2012:95). A qualitative research approach is applicable to researchers who need to examine the quality of human behaviour, making sense of and interpreting the meanings that participants attach to their experiences (Austin & Sutton 2014:436). The design of the study enabled the researcher to explore the resilience of young adults living with mental illnesses, as well as their ability to establish social networking and seek social support.

### Study setting

The study was conducted in a low-resource area located on the peripheries of the city of Tshwane in what was formerly called ‘townships’. Low-resource settings are characterised by limited finances to cover healthcare costs on an individual or societal basis, leading to limited access to medication, equipment, supplies and devices and less developed infrastructure, including transportation (Washington University 2014). The ‘city of Tshwane’ refers to the City of Tshwane Metropolitan Municipality in Pretoria, Gauteng province. Pretoria is the administrative capital city of the Republic of South Africa (South African Government 2020). In this city, mental healthcare services are provided through a provincial hospital on the south-western side of the city and two other private hospitals, as well as through provincial clinics, City of Tshwane Municipality clinics, and public and private rehabilitation centres.

### Study population and sampling

The researcher approached five clinics offering mental healthcare services in the city of Tshwane; however, only two were willing to assist. With assistance from the mental healthcare nurses, the researcher identified those individuals who fit the inclusion criteria and were willing and able to give consent and participate in the study. These included young adults aged between 18 and 34 years living with mental illnesses, residing and utilising the mental health clinics in the city of Tshwane. Purposive sampling was used to recruit 21 participants between March and June 2018. However, four participants withdrew from the study without giving a reason.

Young adults living with schizophrenia, mood disorders, anxiety and depression who were stable on treatment were included, whilst young adults with substance abuse, intellectual disability and those who were not in touch with reality and were unable to give informed consent were excluded from the study. Data collection ceased after the 10th participant, when no new codes were generated, suggesting that the researcher had reached data saturation (Majid et al. 2018:67). According to Creswell (2014), data saturation is reached when no new information is discovered in the data during the data analysis or there is enough information to replicate the study. This is when the ability to obtain additional new information has ended and when further coding is no longer feasible.

### Data collection

Data gathering was done through self-report using face-to-face semi-structured interviews. An interview guide was used to guide the interview. An interview guide was designed with the help of experts and was pretested with the first two interviews to ensure that the questions elicited appropriate responses and confirmed the reliability of the data collection instrument. The interviews were carried out in English, which was a preferred language of communication for all participants. The Robertson Cooper model of resilience guided the questions and probing during the interviews (International Council of Nurses 2016:30). Face-to-face interviews allowed the researcher to collect data on individuals to explore sensitive issues of relationships and support that involved personal histories, as well as the individuals’ perspectives on resilience; this was useful particularly because the researcher was aware of exploring a sensitive topic (Family Health International 2005:2). Other advantages of using interviews in this study included that the researcher was able to clarify questions where there were misunderstandings, the researcher was able to observe some non-verbal behaviours and mannerisms, and the researcher was able to obtain in-depth responses (Brink, Van der Walt & Van Rensburg 2018:153). Questions related to the participants’ sense of confidence, social support and ability to establish social networks, purposefulness and adaptability were included in the interview guide, with additional probes posed to the participants. The interviews were recorded using an audio-recording device, and reflective notes were taken during the interviews.

To ensure rigour, the researcher used Lincoln and Guba’s criteria of credibility, dependability, confirmability and transferability (Lincoln & Guba 1986). According to Forero et al. (2018:3), the purpose of dependability is to ‘establish confidence that the results are true, credible and believable’. To ensure credibility, the researcher spent more than 12 weeks with the setting and participants, and to pretest the interview guide, interviews were conducted with two participants. The researcher further developed a detailed draft of the study protocol and a detailed track record of the data collection process, and an independent coder was also used to code the data and then compare and discuss the codes with the researcher in order to ensure dependability (Forero et al. 2018:3). A reflexive journal was also kept, ensuring that the results would be confirmed by other researchers (Forero et al. 2018:3). Transferability was ensured by purposefully selecting participants who fit the criteria for inclusion in the study and ensuring that data were collected until saturation was reached.

### Data collection procedure

The researcher visited two clinics that had already indicated their availability for the research to proceed. Potential participants were identified from those clinics during the days that they had an appointment with the doctor. The purpose of the study and the procedure involved in the participation were explained by the researcher to the potential participants. Those who were willing to participate were given an information leaflet to further familiarise themselves with the research objectives and procedure before written consent was obtained. After informed consent had been obtained, the research participants were interviewed on the clinic premises in a separate private room after their appointments with the doctors. Each interview lasted for 30–45 min. Only one interview was conducted per session as the researcher did not want to disrupt the treatment flow. The interviews were later transcribed verbatim to begin the data analysis. Data were collected until saturation was reached.

### Data analysis

The data were transcribed verbatim from audiotapes to paper. Using Tesch’s approach to content analysis, the verbatim transcriptions were analysed until data saturation was reached. Tesch’s approach involves categorising verbatim transcriptions into themes for analysis (Creswell & Creswell 2018:192). The transcriptions were carefully read, making notes of ideas that emerged from the data whilst arranging similar topics into groups. The researcher then abbreviated the topics as codes and wrote the codes next to the appropriate segments of text, using the most descriptive wording for the topics and then grouping together topics that were related to each other. The data material belonging to each category was put together in one place, and a preliminary analysis was performed. The organisation of data was observed to check whether new categories or codes emerged. Recoding of the data was done where necessary.

## Ethical considerations

The ethical standards for nurse researchers and fundamental research principles serve as a framework for nurses conducting and participating in research; therefore, the research was based on the ethical standards for nurse researchers (Brink et al. 2018:34).

### Approval from the relevant research committee

The Departmental Research and Innovation Committee, Faculty Higher Degree Committee, Faculty Research Ethics Committee of the Tshwane University of Technology (FCRE 2017/10/08 [SCI] [2]), and the Tshwane Research Committee (GP_201711_015) approved the study and granted permission to the researcher to carry it out in the community.

### The right to self-determination

Informed consent was obtained from each participant before data collection could commence. Transparency was upheld in terms of the objectives of the research, the type of data to be collected, the method of data collection and the benefits of the research. The potential respondents were approached by the researcher on the day of follow-up at the clinic, where they were invited to take part in the study after a thorough explanation and an information leaflet had been provided.

### Principle of beneficence

The researcher ensured the well-being of all participants, who have a right to protection from discomfort and harm – be it physical, psychological, emotional, spiritual, economic, social or legal.

### Principle of justice

The researcher treated all participants fairly and equally during the study. The researcher ensured that there was no victimisation or loss of benefits for those participants who refused to participate in the study or those who withdrew their participation from the study.

### Confidentiality and anonymity

The researcher ensured confidentiality and anonymity through the protection of participants’ identities. The researcher ensured the privacy, worth and dignity of the participants during the study by conducting the interviews with participants in a private area with minimal disturbances. The researcher used fictitious names when reporting to ensure that no link could be made between the individual identities of the participants and the research reports and publications generated from the results.

## Findings

### Demographic information

Table 1 presents the demographic information of all participants, including their age, gender, nature of illness, highest educational level achieved, employment status and relationship status. The participants were aged between 19 and 34 years. Seven participants were male, and the three others were female. In terms of the highest educational level achieved, two participants had diplomas and four participants had completed grade 12. None of the participants were married, but four were in relationships. The participants included in the study were living with mental illness such as anxiety disorder, depression, schizophrenia, bipolar and epilepsy. Although epilepsy is not classified as a mental illness in the literature, people living with epilepsy may experience changes in personality, display extreme emotional changes or exhibit behaviours that are not considered socially acceptable, leading to them being mental healthcare users (Sadock, Sadock & Ruiz 2015:723).

**TABLE 1:** Demographic information.

Participant number	Name	Gender	Age	Illness	Education	Employment	Relationship status
1	Mahlatse	Male	31	Depression	Grade 10	Unemployed	Single
2	Lehlogonolo	Male	19	Anxiety	Grade 11	Scholar	Single
3	Tsietsi	Female	34	Schizophrenia	Diploma	Unemployed	Single
4	Paulus	Male	34	Schizophrenia	Grade 12	Unemployed	Relationship
5	Dorah	Female	29	Epilepsy	Grade 10	Self-employed	Single
6	Beauty	Female	28	Depression, bipolar	Grade 12	Employed	Relationship
7	David	Male	29	Schizophrenia	Diploma	Part-time	Relationship
8	Lebogang	Male	23	Depression	Grade 12	Scholar	Single
9	Jeffrey	Male	32	Schizophrenia	Grade 12	Unemployed	Single
10	Thabo	Male	27	Schizophrenia	Grade 11	Unemployed	Relationship


### Social support

During the data analysis, multiple themes emerged, including *social support, adaptability, purposefulness* and *confidence*. However, for the purpose of this article, only one theme (i.e. *social support*) and the three categories that emerged under it are discussed (see Table 2).

**TABLE 2:** Social support.

Theme	Category	Subcategory
Social support	Poor relationship with family	Lack of support Loneliness Alienation
	Good relationship with family	Supportive relationship Family involvement Financial support Therapeutic relationship
	Good relationships with friends	Spending time together Understanding and empathy Tolerance and compassion

The theme of social support emerged during analysis of the responses to a question that aimed to explore the participants’ ability to seek and secure social support from effective networks and build good relationships to help them overcome adverse situations. Questions relating to the participants’ relationships with their families and friends were posed. The participants reflected on their relationships with their families and friends, and some even reflected on their relationships with colleagues. Categories that emerged from the theme were *poor relationship with family, good relationship with family* and *good relationship with friends*.

#### Poor relationship with family

Under the category of *poor relationship with family*, the participants reported a lack of support, loneliness and alienation from family members. One participant reported a lack of support that led to feelings of loneliness. It appears that those participants who had poor relationships with their families also lacked support from them. The narratives to support this subcategory are as follows:

‘Like, when I do something useful or looking for their [*family*] help when I have problems … maybe in a relationship or anything like that, you see? There is no one that stands by me; I feel like I am alone in this world, which is true, you see?’ (Mahlatse, 31 years old, depression)

‘I am on my own, I do everything by myself. No one at home helps me.’ (Dorah, 29 years old, epilepsy)

#### Good relationship with family

Some participants reported that they had good relationships with their families. Participants mentioned supportive relationships, involvement in their treatment, financial support and therapeutic relationships with their families. Narratives to support this category are as follows:

‘They are supportive … sometimes my sister comes with me to the clinic, for follow-up and check-ups.’ (Paulus, 34 years old, schizophrenia)

‘My sister used to take me to the doctor’s appointment, and she would sometimes fetch my treatment.’ (Beauty, 28 years old, bipolar and depression)

‘Well, it’s amazing, and I’m grateful to have them in my life. They are very supportive and help me with my studies.’ (Lebogang, 23 years old, depression)

‘It is okay; if I want something, they give me money; they are supportive.’ (Tsietsi, 34 years old, schizophrenia)

#### Good relationship with friends

Participants reported that they spent most of their time with their friends, and some described their relationship with friends as understanding and empathetic, whilst other friends were seen as tolerant and compassionate. Narratives to support this category are as follows:

‘We are very close; it’s people I actually grew up with. So, we understand each other very well.’ (Lebogang, 23 years old, depression)

‘They are there for me when I need people to spend time with.’ (Lehlogonolo, 19 years old, anxiety)

‘They are supportive; even when I’m sick, they come to visit me. They pray with me, and they have good words that encourage me.’ (Tsietsi, 34 years old, schizophrenia)

‘They treat me with respect; they do not isolate me.’ (Paulus, 34 years old, schizophrenia)

The participants described their relationships with friends as good; they also mentioned that their friends were encouraging, did not isolate them and spent time with them.

## Discussions

Half of the participants in this study were unemployed. There has also been a general increase in the unemployment rates in South Africa (StatsSA 2019). Unemployment has been associated with poor general health and some poor health behaviours, such as smoking, cannabis use and harmful drinking habits (Carlin et al. 2011:21). Furthermore, unemployed people are twice as likely to experience anxiety and depression, more than twice as likely to engage in harmful consumption of alcohol and smoking, and five times as likely to use cannabis (Carlin et al. 2011:7). The longer unemployment goes on, the more social networks are lost (Carlin et al. 2011:21), with a loss of an important tenet of resilience (Blunt 2016). Unemployment is also associated with experiences such as stigma, anger and frustration, and loss of social roles Guintoli et al. 2011:2).

In a cross-sectional study examining the association and interaction between education and physical and mental health with unemployment in early, mid, and late work life, Van Zon et al. (2017) found that low educational achievement and poor physical and mental health exacerbate each other’s impact on unemployment. The study concluded that addressing unemployment may account for the physical and mental health status of adults during some phases of their lives (Van Zon et al. 2017). Although age is also a significant predictor of resilience, especially in emotional regulation in the general population (Gooding et al. 2012:268), in this study age did not have any significance. Thus, social support is a more important predictor of resilience than age.

Studies were conducted on the interaction between low educational achievement with health outcomes, health-seeking behaviours, health disparities and unhealthy behaviours. However, there are no previous studies that examined any form of relationship between educational achievement and resilience in young adults living with mental illness. In contrast, other studies concluded that low educational achievement and poor physical and mental health exacerbate each other’s impact on unemployment (Van Zon et al. 2017). This study found that the common traits amongst those participants with resilience were good social bonds with family and friends and a higher educational achievement compared to the others. These participants reported good relationships with their families and friends. The two participants who were not resilient, on the contrary, had some similarities, such as lower educational achievement compared with the rest of the participants and poor relationships with their families. Van Zon et al. (2017) showed that low education and mental illness were associated with unemployment, whilst unemployment was associated with a loss of social networks (Carlin et al. 2011:21), poor health and harmful health behaviours.

Social support is about building good relationships with others and seeking support when needed (International Council of Nurses 2016:31). When it comes to resilience, having this network allows people to overcome difficult situations rather than trying to cope on their own (Blunt 2016). Robertson Cooper identified five types of social support (Blunt 2016): *emotional* – reassurance and support; *esteem* – showing encouragement; *network* – a feeling of social connection; *tangible* – assisting with material help; and *informational* – providing facts and advice. The importance of the support provided by family, friends or networks, based upon shared cultural, economic or recreational interests, is strongly emphasised by Buikstra et al. (2010) as a foundation of both community and individual resilience. A supportive social network helps individuals to cope during hard times, and positive and caring individuals strengthen their networks (Buikstra et al. 2010).

Social support is important for maintaining physical and psychological health, and the harmful consequences of poor social support in mental illness are well documented (Ozbay et al. 2007:35). Southwick, Vythilingam and Charney (2005:256) also support this by stating that social support is essential for individuals to maintain good physical and mental health. Lack of support from family, a sense of alienation and loneliness meant that participants experienced challenges in securing social support and establishing social networks, which are essential constituents of resilience (Blunt 2016). Research has also indicated that threats to social connectedness, such as rejection and loneliness, trigger neurobiological systems associated with mental instability (Southwick et al. 2016:78). Those resilient participants also reflected tolerance, compassion, understanding and empathy from their friends and families.

Good social support has been identified as having a positive impact on moderating and enhancing resilience to stress in patients with mental illnesses (Ozbay et al. *2*007:35). Those participants who reported that they had good relationships with their families and friends expressed support, family involvement in their treatment regimens and financial support. Tlhowe, Du Plessis and Koen (2017:33), in their study about the power of families to limit relapse in mentally ill family members, note family involvement in the daily activities of patients with mental illnesses as a strength that contributes to limiting relapses.

Loneliness and alienation were identified as traits that indicated the participants’ lack of resilience in this study. Loneliness or isolation may be experienced because of an individual’s departure from society or exclusion from the community, whilst alienation is seen as an interpersonal phenomenon resulting from the lack of social acceptance towards mental illness (Erdner et al. 2005). An alienated person does not have any sense of belonging and love and remains isolated and estranged (Anju 2015). This condition emerges as a result of a lack of capacity to fit into the social structure, unfulfilled expectations and poor mental health. Therefore, people living with mental illnesses who lack social support are likely to find it difficult to become resilient.

### Strengths and limitations

In the beginning of the study, the researcher identified five clinics that offered mental healthcare services where data could be collected; however, because of a lack of interest and lack of clear protocol in terms of obtaining ethical clearance to conduct research in these facilities, the researcher ended up with only two clinics where data could be collected. The findings emanating from this study may also not be generalised to other contexts.

### Implications and recommendations

Based on the findings from this study, recommendations emerged for nursing practice, nursing education and nursing research. Mental healthcare nurses should include the families of the mental healthcare users during psychotherapy to increase awareness, harness support from significant others and enhance resilience. Community-based mental health programmes should assist mental healthcare users to blend into their communities well and enhance resilience. Furthermore, research has to assess the feasibility of incorporating skills to enhance adaptability, purposefulness, self-esteem, social support and networking amongst mental healthcare users to enhance resilience.

## Conclusion

The main objective of this research study was to explore the social support of young adults living with mental illnesses in the city of Tshwane, Gauteng Province, South Africa. The results indicated that the presence of social support from family and friends led to participants being resilient in the face of adversity. Recommendations to facilitate resilience amongst young adults living with mental illnesses were outlined. This study also provided a direction for future research and mental health education to include the tenets of resilience in an effort to help young adults living with mental illnesses to build and sustain resilience.
